# Monitoring Repair of UV-Induced 6-4-Photoproducts with a Purified DDB2 Protein Complex

**DOI:** 10.1371/journal.pone.0085896

**Published:** 2014-01-28

**Authors:** Matija Dreze, Anne S. Calkins, Judit Gálicza, Daniel J. Echelman, Mathew R. Schnorenberg, Gillian L. Fell, Shigenori Iwai, David E. Fisher, David Szüts, J. Dirk Iglehart, Jean-Bernard Lazaro

**Affiliations:** 1 Department of Cancer Biology, Dana-Farber Cancer Institute, Boston, Massachusetts, United States of America; 2 Department of Surgery, Brigham and Women's Hospital, Boston, Massachusetts, United States of America; 3 Institute of Enzymology, Research Centre for Natural Sciences, Hungarian Academy of Sciences, Budapest, Hungary; 4 Division of Chemistry, Graduate School of Engineering Science, Osaka University, Osaka, Japan; 5 Cutaneous Biology Research Center, Massachusetts General Hospital, Charlestown, Massachusetts, United States of America; Chang Gung University, Taiwan

## Abstract

Because cells are constantly subjected to DNA damaging insults, DNA repair pathways are critical for genome integrity [1]. DNA damage recognition protein complexes (DRCs) recognize DNA damage and initiate DNA repair. The DNA-Damage Binding protein 2 (DDB2) complex is a DRC that initiates nucleotide excision repair (NER) of DNA damage caused by ultraviolet light (UV) [2]–[4]. Using a purified DDB2 DRC, we created a probe (“DDB2 proteo-probe”) that hybridizes to nuclei of cells irradiated with UV and not to cells exposed to other genotoxins. The DDB2 proteo-probe recognized UV-irradiated DNA in classical laboratory assays, including cyto- and histo-chemistry, flow cytometry, and slot-blotting. When immobilized, the proteo-probe also bound soluble UV-irradiated DNA in ELISA-like and DNA pull-down assays. *In vitro*, the DDB2 proteo-probe preferentially bound 6-4-photoproducts [(6-4)PPs] rather than cyclobutane pyrimidine dimers (CPDs). We followed UV-damage repair by cyto-chemistry in cells fixed at different time after UV irradiation, using either the DDB2 proteo-probe or antibodies against CPDs, or (6-4)PPs. The signals obtained with the DDB2 proteo-probe and with the antibody against (6-4)PPs decreased in a nearly identical manner. Since (6-4)PPs are repaired only by nucleotide excision repair (NER), our results strongly suggest the DDB2 proteo-probe hybridizes to DNA containing (6-4)PPs and allows monitoring of their removal during NER. We discuss the general use of purified DRCs as probes, in lieu of antibodies, to recognize and monitor DNA damage and repair.

## Introduction

Response to DNA damage caused by genotoxic stress involves recognition of the damage and subsequent repair. Distinct DNA repair pathways have evolved to respond to different categories of DNA damage. Specific DNA damage recognition protein complexes (DRCs) recognize and bind the various lesions found in DNA to initiate their cognate DNA repair pathway. Failure or delay to repair DNA leads to accumulation of mutations and can result in disease, including cancer [1], [5].

UV light is a pervasive genotoxin that can cause skin cancer. Upon reaching DNA, UV light predominantly causes intra-strand crosslinks of two adjacent pyrimidines, causing cyclobutane pyrimidine dimers (CPDs) and 6-4-photoproducts [(6-4)PPs] [6], [7]. Both types of lesions are repaired by the nucleotide excision repair pathway (NER), albeit on different time scales. Recognition of UV damaged DNA by the DNA Damage Binding protein 2 complex (DDB2) is necessary for the timely completion of global genome repair (GGR) of UV lesions by NER *in vivo* [8]–[11]. Several results obtained with *in vitro* assays and from genetic evidence have shown DDB2 binds both types of lesions, but has a higher affinity for (6-4)PPs compared to CPDs [12]–[15]. In addition, a crystal structure of DDB2 bound to (6-4)PPs or CPDs have been resolved [15], [16].

The DDB2 protein complex is constituted of several sub-complexes, and does not require prior activation to recognize DNA damaged by UV light. Before damage, the complex is stabilized by the presence of the COP9 signalosome sub-complex [17]. Damage recognition involves dissociation of the COP9 sub-complex, ubiquitylation of DDB2 by the DDB1-Cul4 ubiquitin ligase sub-complex, and subsequent degradation of DDB2 [17]. Degradation of DDB2 allows displacement of the recognition complex from the lesion, and initiation of repair [18], [19]. Repair is performed in sequential steps by several protein complexes. These steps include unwinding of DNA, excision of a single strand fragment of 24–32 nucleotides containing the lesion, and gap filling using the undamaged strand as template [20]–[22]. Mutations in seven well characterized NER genes (XPA to XPG), including DDB2 (XPE), result in Xeroderma Pigmentosum (XP), a recessive inherited syndrome characterized by heightened UV-sensitivity, neurological abnormalities, and an increased susceptibility to develop skin cancers [23], [24].

**Figure 1:**
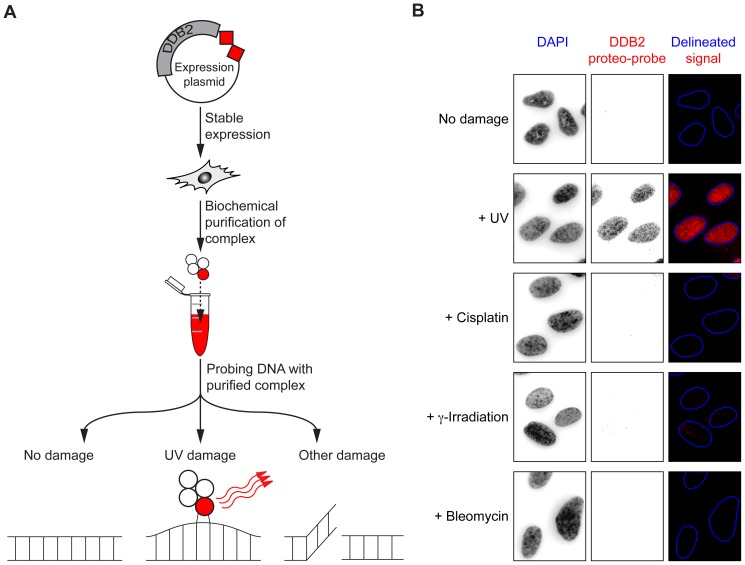
A purified DDB2 protein complex can be used to detect UV-induced DNA damage. (**A**) Experimental strategy to prepare the DDB2 proteo-probe. (**B**) Signal obtained by hybridization of the DDB2 proteo-probe onto fibroblasts with or without damaging treatments. Hybridized DDB2 proteo-probe is revealed by anti-HA immunofluorescence. Nuclei are visualized by DAPI staining. Nuclei are delineated based on DAPI staining and using CellProfiler [26].

We hypothesized the purified DDB2 complex would carry the recognition activity of the endogenous complex, and could be employed like an antibody in immune-based techniques (Figure 1A). We call such a purified complex used as a probe a “proteo-probe”. We found the DDB2 proteo-probe binds preferentially to (6-4)PPs rather than CPDs *in vitro*. We observed the DDB2 proteo-probe hybridizes to nuclei of fixed UV-irradiated cells, and allows monitoring of repair. The observed kinetic of repair corresponds to the repair of (6-4)PPs. We conclude we created a probe specific for 6-4-photoproducts.

## Materials and Methods

### Cell lines and cell culture

Human BJ1 newborn foreskin fibroblasts (American Type Culture Collection, Manassas, VA) and HeLa S3 (Sigma-Aldrich, St. Louis, MO) were maintained at 37°C, 100% humidity, 5% CO_2_ in Dulbecco's modified Eagle's medium supplemented with 10% fetal bovine serum (v/v), penicillin (10^5^ units/l) and streptomycin (100 mg/l; all reagents purchased from Life Technologies, Carlsbad, CA).

### Primary antibodies

Mouse monoclonal anti-FLAG conjugated to horseradish peroxidase (1∶1,000; clone M2; Sigma-Aldrich).
Purified mouse monoclonal anti-HA (1∶200; clone 16B12, Covance, Princeton, NJ).
Rabbit anti-Cullin4A (1∶500; Cell Signaling Technology, Beverly, MA).
Rabbit anti-DDB1 (1∶500; Santa Cruz Biotechnology, Santa Cruz, CA).
Rabbit anti-CSN5 (1∶500; Sigma-Aldrich).
Purified mouse monoclonal anti-cyclobutane pyrimidine dimer (1∶2,000; Kamiya Biomedical, Seattle, WA).
Mouse monoclonal anti-(6-4)-photoproducts (1∶400; Cosmo Bio Co., LTD., Japan).

### Affinity purification

DDB2-FLAG-HA was purified from a HeLa S3 cell line previously published [17]. This cell line expresses the DDB2 open reading frame fused to a FLAG-HA tag. We performed affinity purification as described earlier [25]. Briefly, we washed cells in phosphate buffer saline (PBS, 10 mM, pH = 7.4), then treated cells with lysis buffer (40 mM Tris-HCl [pH = 8], 200 mM NaCl, 10% glycerol, 2 mM EDTA, 0.4% NP40) supplemented with a protease inhibitor cocktail (Roche Applied Sciences, Indianapolis, IN), for 30 minutes at 4°C. The cell lysate was cleared by centrifugation at 25,000× *g* for 30 min at 4°C. The supernatant was then incubated for 4 hours at 4°C with M2 anti-FLAG antibody-coated agarose beads (Sigma-Aldrich). We eluted the complex from the beads by incubation with excess FLAG peptide (Sigma-Aldrich) for 2 hours at 4°C and recovered the eluate by centrifugation through a Bio-Spin chromatography column (Bio-Rad Laboratories, Hercules, CA).

### Silver staining and immuno-blotting

We resolved the DDB2 protein complex in a NuPAGE 4–12% gel (Life Technologies) and analyzed the complex by silver staining or by immuno-blotting with indicated antibodies. Silver staining was performed with a SilverQuest Kit (Life Technologies). We visualized immuno-blots with Supersignal chemi-luminescence reagents (Pierce, ThermoScientific, Rockford, IL), and a luminescence image analyzer LAS-4000 mini (Fujifilm, Edison, NJ).

### *In situ* fluorescence

Cells were grown on glass coverslips, or on multi-well glass slides (Electron Microscopy Sciences, Hatfield, PA), or using the DropArray system and Liquid Lid Sealing Fluid (Curiox Biosystems Inc., San Carlo, CA). To perform “fixation/extraction”, we applied methanol (−20°C) to cells and incubated them at room temperature for 10 minutes. We then serially re-hydrated cells in methanol-PBS (50, 25, 12.5, 6.25, 3.12, 1.56, and 0% methanol). To block non-specific sites, fixed cells were incubated in PBS-BSA (PBS, 0.3% bovine serum albumin, 0.1% sodium azide). We applied the DDB2 proteo-probe diluted in PBS-BSA to cells for 30 minutes at 37°C. We removed un-hybridized DDB2 proteo-probe with two washes in PBS and labeled the hybridized proteo-probe for one hour at 37°C with 5 µg/ml anti-HA antibody diluted in PBS-BSA. After two washes in PBS, we incubated cells for 30 minutes at 37°C with 6.67 µg/ml goat anti-mouse antibody coupled to Alexa fluor488 fluorochrome (Life Technologies) diluted in PBS-BSA. After two washes in PBS, and one wash in purified water, we mounted coverslips in hardset Vectashield medium containing DAPI (Vector Laboratories, Burlingame, CA).

For immuno-fluorescence against CPDs and (6-4)PPs, after fixation, chromatin DNA was denatured by treatment with concentrated hydrochloric acid. When using the anti-CPD antibody, after methanol fixation and rehydration of cells, we sequentially incubated cells at room temperature with PBS (10 minutes), purified water (10 minutes), 4N hydrochloric acid (5 minutes), purified water (10 minutes), and PBS (10 minutes) before blocking with PBS-BSA and immuno-fluorescence. The anti-(6-4)PP antibody was used according to the manufacturer's instructions. Briefly, cells were fixed with 4% formalin and extracted with 0.5% Triton-X100. Chromatin DNA was denatured with 2N hydrochloric acid for 30 minutes, and cells were washed five times in PBS. After non-specific signal was blocked with PBS-BSA, cells were treated for immuno-fluorescence.

### Image acquisition and processing

We visualized fluorescence on an upright microscope (Imager.M2, Zeiss, Germany) equipped with an HXP 120C light source. We photographed cells with an AxioCam MRM camera coupled with a 10×/0.45 plan-APOCHROMAT, or 63×/1.4 oil plan-APOCHROMAT objective. The imaging platform was controlled using the Axiovision 4.8 software (Zeiss). For each field of view we acquired five images in a vertical stack (*z*-stack): one image in the focal plane, plus two images above and two images below. Within a *z*-stack, images taken with the 10×, or with the 63× objective were separated by 1.7 µm, and 0.3 µm, respectively. We processed images using the CellProfiler imaging platform [26]. We assembled “projected images” by combining the five images of a *z*-stack. This strategy eliminates signals that vary from one layer of the *z*-stack to another (non-specific signal). For each field of view, we quantified fluorescence signals in projected images and obtained: (i) the number of nuclei, (ii) the fluorescence signal intensity for each nucleus, (iii) the number of foci, and (iv) the fluorescence signal intensity outside nuclei.

For cytochemistry and histochemistry experiments, we acquired images on a BX41 microscope coupled to a Qcolor5 camera (Olympus, Center Valley, PA).

### DNA damaging treatments (Figure 1B)

We treated BJ1 fibroblasts with one of several genotoxins before fixation: 20 J/m^2^ UV-C at 254 nm using a StrataLinker 2400 (Stratagene, Agilent Technologies, Santa Clara, CA), 100 µg/ml of cisplatin for two hours (Sigma-Aldrich), 10 ng/ml of bleomycin for one hour (Sigma-Aldrich), or 30 Gray of ionizing radiation.

### DNase treatment (Figure 2B)

We treated BJ1 fibroblasts with 3×10^−3^ Kunitz units of DNaseI diluted in RDD buffer (Qiagen, Germany) for 10 minutes at RT prior to blocking with PBS-BSA and DDB2 proteo-probe fluorescence.

### Competition experiment (Figure 2C)

We irradiated plasmid DNA with 300 J/m^2^ UV-C. Prior to hybridization onto cells, we incubated the DDB2 proteo-probe with indicated amounts of untreated or UV-treated plasmid DNA at RT for 30 minutes.

### *In vitro* DNA pull-down assay (Figure 2D)

We obtained DNA oligonucleotides containing two CPDs, or two (6-4)PPs, or no lesion at all. The lesions were located on opposite strands in a staggered arrangement, 28 base pairs apart. These oligonucleotides were ligated into the pQ1 vector [27]. The resulting plasmids and the lesion-free pQ1 control were submitted to restriction-digest to completion with the *Dpn*I and *Sca*I restriction enzymes. We obtained the DDB2 proteo-probe as described in “Affinity purification”, with the difference that the purified complex was not eluted from the M2 anti-FLAG antibody-coated agarose beads.

We performed DNA pull-downs with the DDB2 proteo-probe bound to M2 anti-FLAG antibody-coated agarose beads and the plasmid restriction DNA fragments containing either CPDs, or (6-4)PPs, or no lesion. Bound DNA was isolated from the beads, and was used as template for qPCR with primer pairs designed against the lesion-containing fragment (forward: 5′-ATCGCCCTGATAGACGGTTT-3′, reverse: 5′-CCGAGATAGGGTTGAGTGTTG-3′) and against a similar sized lesion free restriction fragment of pQ1 (forward: 5′-GAACCAACAAATGTCCAAACCG-3′, reverse: 5′- AACAAGGAGGTAAATGGGGAGTG-3′) [28].

### UV micro-irradiation (Figure S3A)

We placed a micro-porous isopore membrane (pores of 5 µm in diameter, Millipore, Cork, Ireland) between cells grown on glass coverslips and the UV source, and irradiated covered cells with 300 J/m^2^ UV-C.

### Histochemistry (Figure S4A)

We irradiated shaved backs of living C57BL/6 mice with 2,500 J/m^2^ UV-B. We embedded skin punch biopsies in OCT mounting medium, and processed tissues for histochemistry. Briefly, we fixed 5-micron thick sections placed on plus glass slides in ice-cold methanol-acetone (1∶1) for 30 minutes. We serially re-hydrated tissue sections in methanol-acetone/PBS (50, 25, 12.5, 6.25, 3.12, 1.56, and 0% methanol-acetone). Next, we incubated slides in a solution of 3% hydrogen peroxide for 15 minutes, then in PBS supplemented with 3% BSA for two hours. We applied the DDB2 proteo-probe diluted in PBS-BSA to tissue sections, for 60 minutes at 37°C then washed samples in PBS. We labeled hybridized proteo-probe overnight at 4°C with 4 µg/ml anti-FLAG-HRP in PBS-BSA. After washes in PBS, we stained samples with 3,3′-diaminobenzidine for 7 minutes. We washed samples in purified water, counter-stained with hematoxylin, and dehydrated in successive solutions of ethanol and xylene. We mounted samples with coverslips in Clearmount medium (Life Technologies).

### Cytochemistry (Figure S4B)

When performing cytochemistry, fixation, re-hydration, blocking and incubation with the DDB2 proteo-probe were identical to those of the *in situ* fluorescence protocol. We then labeled the hybridized proteo-probe with 4 µg/ml anti-FLAG-HRP antibody diluted in PBS-BSA. After two washes in PBS, we stained the samples with 3,3′-diaminobenzidine for 3 minutes. After one wash in purified water, we mounted coverslips in Clearmount Medium (Life Technologies).

### ELISA-like assay (Figure S4C)

In a maxisorp 96-well microtiter plate (Thermo Scientific, Rochester, NY), we adsorbed 50 ng of anti-HA antibody per well overnight at 4°C in PBS, incubated each well in PBS with 1% BSA for 30 minutes at room temperature, washed six times with PBS-Tween 0.05%, then once with lysis buffer. Next, we added the diluted DDB2 proteo-probe for 5 hours at 4°C, washed twice with lysis buffer (described in “Affinity purification”), added 100 ng of DNA for 30 minutes at room temperature, followed by three washes with lysis buffer. We quantified captured DNA using Picogreen (Life Technologies).

### Slot-blot (Figure S4D)

We collected cells grown in a 3-cm Petri dish in 1 ml of lysis buffer. Ten percent of the lysate was loaded on a Minifold II slot blot system (Schleicher & Schuell, Keene, NH) transferred to a nitrocellulose membrane (0.45 µm, Bio-Rad Laboratories) by vacuum suction and dried overnight at room temperature. We incubated the membrane with PBS-BSA-0.05% Tween (PBT) for 30 minutes. We applied the DDB2 proteo-probe for 30 minutes, washed the membrane twice in PBT, labeled it with 1 µg/ml of anti-FLAG-HRP for one hour at room temperature before washing in PBT. We visualized hybridized proteo-probe as described in “Silver staining and immuno-blotting”. After washes, total DNA was stained with methylene blue and photographed.

### Flow cytometry (Figure S4E)

Non-adherent KOPT-K1 lymphoblastic T-cells grown to 2×10^6^ cells/ml were collected by centrifugation, washed in PBS, fixed in 1% paraformaldehyde on ice for 15 minutes, washed twice in PBS, then suspended and stored overnight in ice-cold ethanol. We washed cells in PBS, applied 30 J/m^2^ UV-C and processed samples as described in “*In situ* fluorescence” before analysis by flow cytometry.

### Statistical analyses

All data were analyzed, fitted, and plotted using GraphPad Prism version 6.0a for Mac, (GraphPad Software, La Jolla, California, USA, www.graphpad.com). Outliers were identified using the ROUT method (Q = 1%). Statistical significance was calculated using two-sided two-sample Student's *t*-tests, unless otherwise noted. The threshold for significance was chosen at *P*<0.05.

## Results

### Specific detection of UV damage

We hypothesized the biochemically purified DDB2 DRC could be a ready-to-use reagent to detect specific DNA damage, and employed to monitor repair in lieu of antibodies (Figure 1A). The composition of the DDB2 complex, obtained by non-denaturing affinity purification of a FLAG-HA tagged DDB2 protein stably expressed in HeLa S3 cells was previously reported [17]. We used these HeLa S3-DDB2-FLAG-HA cells to purify large amounts of the DDB2 complex (Figure S1) and verified the presence of previously reported key components of the DDB2 complex by immuno-blotting (Figure S1). We call this purified multi-protein complex the DDB2 proteo-probe. We tested the recognition activity of the proteo-probe toward DNA damage. BJ1 fibroblasts were subjected to various types of damage and fixed. The diluted DDB2 proteo-probe was applied to fixed cells, instead of a primary antibody, in a classic immuno-fluorescence protocol. To assess whether the proteo-probe hybridized to these cells, we performed immuno-fluorescence against its HA tag. No hybridization was found on untreated cells or cells subjected to cisplatin, bleomycin or ionizing radiation (Figure 1B). In contrast, we observed a strong signal localized in the nuclear region of cells irradiated with UV-C (Figure 1B). We found the DDB2 proteo-probe also hybridized to the nuclei of cells irradiated with UV-B, but not UV-A (Figure S2). It was shown the endogenous DDB2 protein re-localizes at sites of UV damage after irradiation. To understand if the DDB2 proteo-probe indeed hybridized to the very sites of damage, we created localized damage by irradiating cells covered with a micro-porous membrane. After irradiation, cells were fixed, and by cytochemistry we found the proteo-probe hybridizing to regions restricted by the membrane micro-pores inside nuclei (Figure S3A).

**Figure 2:**
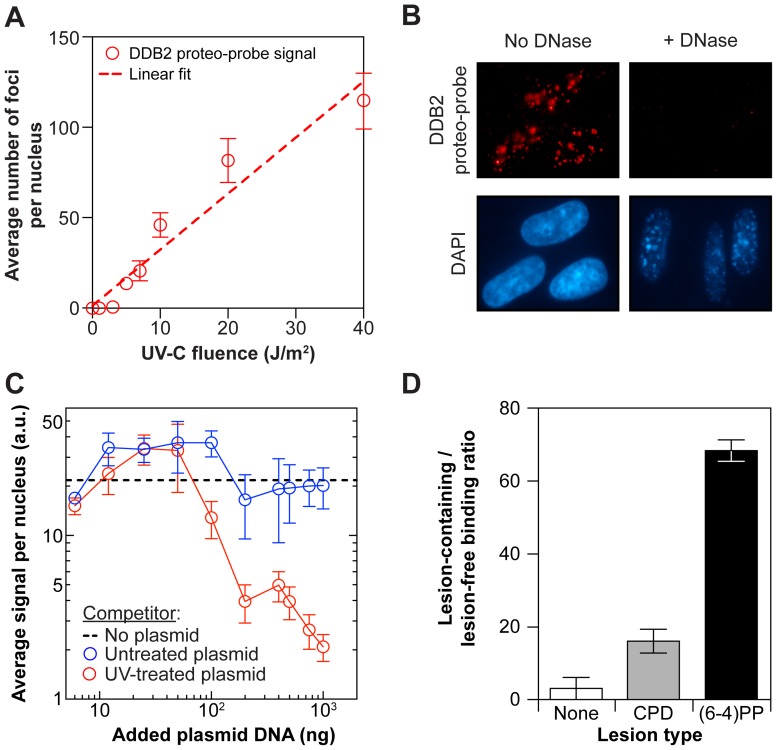
The DDB2 proteo-probe recognizes 6-4-photoproducts *in vitro*. (**A**) The DDB2 proteo-probe signal increases linearly with fluence (J/m^2^). Fibroblasts were irradiated with different doses of UV-C. Each point is an average of three replicas. Each replica represents an average of at least 60 cells. Dashed line: linear fit (R^2^ = 0.94). Error bars: s.e.m. (**B**) The DDB2 proteo-probe signal is DNA-dependent. Fibroblasts were irradiated with UV-C (10 J/m^2^), and untreated or treated with DNase. Nuclei are visualized by DAPI staining. (**C**) The DDB2 proteo-probe signal can be competed with UV-treated plasmid DNA. Fibroblasts and plasmid DNA were irradiated with UV-C (10 J/m^2^ and 300 J/m^2^, respectively). The DDB2 proteo-probe was incubated with plasmid DNA prior to hybridization onto irradiated fibroblasts. Dashed line: no plasmid control proteo-probe signal level. Each point is an average of three replicas. Each replica represents an average of at least 400 cells. Error bars: s.e.m. (**D**) The DDB2 proteo-probe binds preferentially to 6-4-photoproducts [(6-4)PP] over cyclobutane pyrimidine dimers (CPD). The DDB2 proteo-probe was immobilized on agarose beads, and incubated with the DNA restriction fragments of a plasmid containing, or not, a unique lesion [(6-4)PP or CPD]. The average ratio of the amount of lesion-containing over lesion-free DNA fragments bound to the proteo-probe is shown (*n* = 3). Error bars: s.e.m.

We conducted an exposure-response experiment to determine the performance of the proteo-probe within a range of UV doses commonly used. We quantified fluorescence signals per nuclear region using the CellProfiler software [26]. We found both the number of DDB2 proteo-probe foci and the average fluorescence were directly proportional to the UV dose (Figure 2A and Figure S3B, respectively). This suggests a linear relationship between signal and damage, which is in agreement with the positive correlation between UV dose and amount of DDB2 bound to lesions [29]. We wondered if in the experiment shown in Figure 1B, the endogenous DDB2 protein complex interfered with the hybridization of the DDB2 proteo-probe. To immobilize the endogenous DDB2 complex, and prevent its UV-induced re-localization on damage sites, we sequentially: (i) killed cells by fixation, (ii) applied UV irradiation, and (iii) incubated cells with the DDB2 proteo-probe. The intensity of the hybridization signal obtained on cells fixed before irradiation did not appear affected when compared to cells treated in a traditional sequence of irradiation then fixation. This suggests the endogenous DDB2 complex does not interfere with recognition of damage by the DDB2 proteo-probe in a discernable manner under our experimental conditions (Figure S3C).

We assessed the performance of the DDB2 proteo-probe in various types of immuno-chemistry-like assays in which the proteo-probe replaced the traditional primary antibody. We irradiated the back of living mice and processed skin biopsies for histochemistry, or irradiated and subsequently fixed cultured cells for cytochemistry. In both cases, after following standard protocols, we detected the proteo-probe hybridized to the nuclei of damaged cells (Figure S4A and S4B). In addition, the DDB2 proteo-probe adsorbed to a 96-well microtiter plate and tested in an ELISA-like format captured damaged DNA in a UV-dose dependent way (Figure S4C). The probe is also usable in blotting techniques as it hybridized to UV-irradiated purified DNA immobilized to nitrocellulose (Figure S4D). Finally, we could discriminate untreated or UV-irradiated fixed cultured cells by flow cytometry (Figure S4E). Therefore the DDB2 proteo-probe functions in a variety of experimental conditions, and is adaptable to multiple laboratory demands.

### The DDB2 proteo-probe recognizes DNA 6-4-photoproducts

To confirm the signal found *in situ* is indeed DNA dependent, we fixed UV-irradiated fibroblasts and treated them with DNase prior to application of the proteo-probe. The intensity of the DAPI staining greatly decreased after DNase treatment, and the DDB2 proteo-probe staining was completely abrogated (Figure 2B). Next, we incubated the DDB2 proteo-probe with varying amounts of untreated or UV-irradiated plasmid DNA, prior to hybridization onto UV-irradiated fibroblasts. The DDB2 proteo-probe signal remained unaffected by any amount of untreated plasmid, but was drastically reduced by competition with UV-irradiated plasmid DNA, particularly at higher amounts of the competitor (Figure 2C). We conclude the DDB2 proteo-probe recognizes UV-damaged DNA.

Irradiation of DNA with UV-C light produces mostly CPDs and (6-4)PPs. We therefore assessed the recognition of CPDs and (6-4)PPs by the DDB2 proteo-probe. DNA fragments containing either CPDs or (6-4)PPs, or no lesion were incubated with the DDB2 proteo-probe immobilized on agarose beads cross-linked to an anti-FLAG antibody in a pull down experiment. The DNA pulled down by the proteo-probe was isolated then amplified by qPCR. In our experimental conditions, the DDB2 proteo-probe showed preferential binding to DNA fragments containing (6-4)PPs over CPDs (Figure 2D). Altogether, our results strongly suggest the DDB2 proteo-probe hybridizes to UV-damaged DNA, and specifically to foci containing (6-4)PPs.

### Monitoring repair of 6-4-photoproducts with the DDB2 proteo-probe

We wondered if the DDB2 proteo-probe would allow monitoring the repair of (6-4)PPs by *in situ* fluorescence experiments. To follow repair of damage over time, BJ1 fibroblasts were irradiated with 10 J/m^2^ of UV-C, and fixed at various time points after damage. We compared signals obtained with the DDB2 proteo-probe, anti-CPD, and anti-(6-4)PP antibodies. Since the anti-CPD and anti-(6-4)PP antibodies were raised against purified single-stranded DNA oligonucleotide containing a single lesion, strong chromatin denaturing conditions are necessary to uncover epitopic UV damaged cellular DNA [11]. Therefore for immunofluorescence analysis with antibodies against CPDs and (6-4)PPs, we treated fixed fibroblasts with concentrated hydrochloric acid. In contrast, cyto-chemistry with the DDB2 proteo-probe was directly performed on fixed cells.

**Figure 3:**
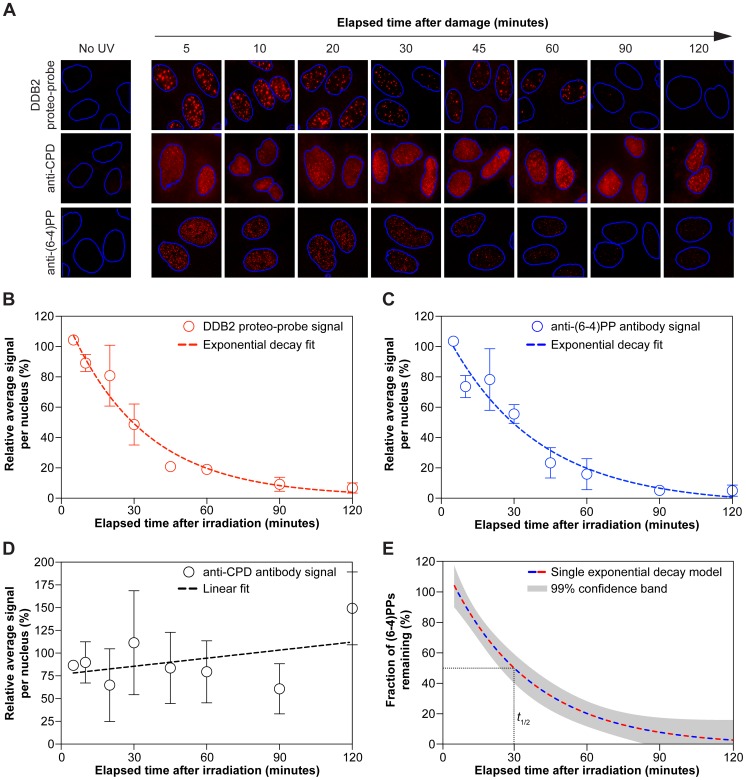
The decrease of DDB2 proteo-probe and 6-4 PP signals over time are nearly identical. (**A**) Typical signals after UV damage observed *in situ* with the DDB2 proteo-probe, an anti-CPD antibody, or an anti-(6-4)PP antibody. Nuclei are delineated based on DAPI staining and using CellProfiler. (**B**) The DDB2 proteo-probe signal decreases exponentially with time. Average signal per nucleus normalized to signal at 5 minutes. Red dashed curve: one phase exponential decay fit calculated with a non-linear least square method (R^2^ = 0.86). (**C**) The anti-(6-4)PP signal decreases exponentially with time. Average signal per nucleus normalized to signal at 5 minutes. Blue dashed curve: one phase exponential decay fit calculated with a non-linear least square method (R^2^ = 0.83). (**D**) The anti-CPD signal remains constant over a two hour period. Average signal per nucleus normalized to signal at 5 minutes. Black dashed line: linear fit on the α-CPD signal (R^2^ = 0.18). (**B**), (**C**), and (**D**): cells were irradiated with UV-C (10 J/m^2^). The average of three replicas is shown. Each replica represents an average of at least 60 cells. Error bars: s.e.m. (**E**) A single one phase exponential decay model summarizes the kinetic of (6-4)PPs removal *in situ*. The single model is based on the decay fits obtained with DDB2 proteo-probe and anti-(6-4)PP data. The grey band represents the area enclosing the true decay curve with 99% confidence. The dotted line indicates the predicted half-life (*t*_1/2_) of (6-4)PPs *in situ* after UV irradiation.

The DDB2 proteo-probe signal, maximal five minutes after UV irradiation, decreased to minimal levels at two hours (Figure 3A, top row). We observed no remarkable fluctuation of the signal beyond the two hour time point (data not shown). An almost identical pattern was observed using the anti-(6-4)PP antibody (Figure 3A, bottom row). In stark contrast, the anti-CPD antibody signal did not substantially change over the two hour period (Figure 3A, middle row). The signal per nucleus obtained with the DDB2 proteo-probe, anti-(6-4)PP and anti-CPD were quantified for each of the time points and analyzed for trends.

We fitted a linear regression model on data obtained with anti-CPD antibodies (Figure 3D). Although the fit to the α-CPD data is rather poor (R^2^ = 0.18), we found the data does not significantly deviate from linearity (*P* = 0.63, Runs test), and the slope of the linear fit does not significantly deviate from the horizontal (*P* = 0.30, *F* test). This analysis supports the conclusion that the anti-CPD signal remains relatively constant over a two hour period.

We then fitted one-phase exponential decay models to the DDB2 proteo-probe, and the anti-(6-4)PP data (Figure 3B and 3C). We determined that both fits are not statistically different from each other, and a single exponential decay model adequately fitted both datasets (extra sum-of-squares *F* test, *P* = 0.9002; R^2^ = 0.85; Figure 3E). These data further support the contention that the DDB2 proteo-probe recognizes (6-4)PPs *in situ*. Under this single model, we can predict half of (6-4)PPs (*t*_1/2_) will be undergoing repair within ∼30 minutes in UV-irradiated cultured cells (Figure 3E).

Altogether, given that the DDB2 proteo-probe preferentially binds (6-4)PP lesions *in vitro*, and that its signal decay over time is nearly identical to the disappearance of (6-4)PPs in UV-irradiated cultured cells, we conclude the DDB2 proteo-probe, a multi-protein complex purified from human cells, allows detection of (6-4)PPs and monitoring of their removal *in situ*.

## Discussion

In this study we demonstrate that a purified DDB2 protein complex (“proteo-probe”) detects UV-damaged DNA in cells and tissues, in various assays. We show that the DDB2 proteo-probe detects 6-4-photoproducts and can be used to follow their repair *in situ*.

### The DDB2 proteo-probe is a ready to use reagent

We obtained the DDB2 proteo-probe by purifying the multi-protein DDB2 complex from the HeLa S3-DDB2 Flag-HA cell line established by Groisman, Polanowska and colleagues [17]. Since all protein subunits in the complex may not be needed for the recognition activity, further studies may identify the minimal set of DDB2 partners required for the assembly of a functional proteo-probe. This might permit production of a DDB2 proteo-probe in bacteria or insect cells. However, HeLa S3 cells can be grown in suspension to industrial quantities, and therefore allow production of large amounts of recombinant proteins. During the course of our work, several batches of DDB2 proteo-probe were prepared and stored at −20°C or −40°C in a solution containing 50% glycerol. The DDB2 proteo-probe was then routinely pipetted from inside a bench-top cooler protection box, not unlike traditional restriction enzymes. In this experimental setting, tested over several years and by multiple users, the various lots of DDB2 proteo-probe were very stable and were used without noticeable loss off activity for at least six months after purification.

### The DDB2 proteo-probe hybridizes to specific regions of chromatin

Despite the fact that UV light was applied homogenously onto entire nuclear areas, the DDB2 proteo-probe signal formed foci within nuclei of irradiated cells. This suggests the access of the proteo-probe to chromatin is restricted to sub-regions, which is in agreement with reports that DDB2 predominantly (80%) binds to highly accessible inter-nucleosomal sites of chromatin in damaged cells [30], [31]. In addition, when cells were killed by fixation to prevent any cellular response, irradiated *a posteriori*, and incubated with the DDB2 proteo-probe, we observed similar focal signals (Figure S3). It is therefore likely the discrete regions of chromatin to which the proteo-probe hybridizes already existed before irradiation, consistent with highly accessible inter-nucleosomal sites. Unlike the DDB2 proteo-probe, the use of anti-(6-4)PPs antibodies requires aggressive chromatin denaturing treatment to unravel naked DNA epitopes. Consequently, anti-(6-4)PPs antibodies have access to more (6-4)PPs than the DDB2 proteo-probe, in otherwise un-exposed sites, possibly within nucleosomes. It is therefore not surprising that we observed a greater number of foci when using anti-(6-4)PPs antibodies.

### The DDB2 proteo-probe allows monitoring NER of (6-4)photoproducts

Our *in situ* experiments suggest the DDB2 proteo-probe recapitulates the recognition activity of the endogenous DDB2 complex toward (6-4)PPs, but not toward CPDs.

The role of endogenous DDB2 in the repair of CPDs *in vivo* has been described using a variety of techniques and genetic approaches [12]–[15]. It was shown DDB2 has a much greater affinity for (6-4)PPs compared to CPDs. In our in situ experiments, the DDB2 proteo-probe did not recognize CPDs (Figure 3; compare panels 3A and 3B to panels 3C and 3D). Furthermore, in DNA pull-down assays the DDB2 proteo-probe bound CPDs but with less affinity than (6-4)PPs (Figure 2D).

According to the most recently published model [32], 90% of (6-4)PPs are excised within two hours after irradiation. Our results are entirely consistent with this model since the data obtained by *in situ* fluorescence with anti-(6-4)PP antibodies and by using the DDB2 proteo-probe show a similar fraction of excised (6-4)PPs two hours after irradiation. Because (6-4)PPs are repaired only by the nucleotide excision repair pathway in human cells, monitoring (6-4)PPs levels over time reflects NER of (6-4)PPs. We anticipate the DDB2 proteo-probe will allow studies of NER activities, without the need for chromatin extraction, and can be used in a variety of traditional cyto- and histo-chemistry protocols with standard cell fixation, *e.g.* methanol fixation.

Using the DDB2 proteo-probe did not show obvious advantages over the anti-(6-4)PP antibody. However, antibodies are only available for a few types of DNA lesions. From the proof-of-principle presented here using the DDB2 protein complex, it is likely specific proteo-probes could be obtained from other purified DNA damage recognition complexes and used to detect specific DNA lesions and monitor their repair.

## Supporting Information

Figure S1 **Analysis of the purified DDB2 protein complex components.** (**A**) Visualization by silver staining of the DDB2 protein complex obtained by FLAG-affinity purification, and resolved by electrophoresis on a polyacrylamide gel. Purified DDB2 DNA damage recognition complex: “DDB2 proteo-probe”. M.W.: molecular weight; kDa: kiloDalton. (**B**) Western blotting analysis of key components of known DDB2 protein sub-complexes. DDB1 and Cullin4A of the ubiquitin ligase sub-complex as well as CSN5 of the COP9 signalosome sub-complex are detected along with FLAG-DDB2.
(PDF)

Figure S2 ***In situ*** **detection of UV-A and UV-B DNA damage with the DDB2 proteo-probe.** The DDB2 proteo-probe detects damage induced by UV-B but not UV-A. Fibroblasts were fixed prior to irradiation with different doses of UV-A or UV-B light. The DDB2 proteo-probe was added to fixed cells following irradiation. Hybridized DDB2 proteo-probe is revealed by anti-HA immunofluorescence. Nuclei are visualized by DAPI staining. One representative nucleus is shown for each experimental condition.
(PDF)

Figure S3 **Characterization of the DDB2 proteo-probe hybridization properties.** (**A**) The DDB2 proteo-probe signal is localized at sites of UV damage. Fibroblasts, uncovered or covered by a micro-porous membrane, were irradiated with UV-C (300 J/m^2^). (**B**) The DDB2 proteo-probe signal increases linearly with fluence. Fibroblasts were irradiated with different doses of UV-C. Each point is an average of three replicas. Each replica represents an average of at least 200 cells. Error bars: s.e.m. (**C**) The DDB2 proteo-probe signal is independent of endogenous proteins. Fibroblasts were irradiated with UV-C (10 J/m^2^), then fixed, or fixed then irradiated. The DDB2 proteo-probe was hybridized following fixation/irradiation.
(PDF)

Figure S4 **The DDB2 proteo-probe can be used in different assay formats.** (**A**) Hybridization of the DDB2 proteo-probe onto frozen sections of irradiated mouse skin. The DDB2 proteo-probe bound to punch biopsies was revealed by HRP-conjugated anti-FLAG immunohistochemistry. (**B**) Irradiated fibroblasts (20 J/m^2^ UV-C) were fixed in methanol. Cytochemistry was done with the DDB2 proteo-probe in place of primary antibody. The hybridized proteo-probe was revealed by HRP-conjugated anti-FLAG. (**C**) The DDB2 proteo-probe retains irradiated plasmid DNA in a manner dependent on the amount of UV in an ELISA-like assay. Equal amount of the DDB2 proteo-probe was adsorbed onto wells of a 96-well microtiter plate. One hundred nanogram of UV-irradiated plasmid DNA was added in each well. Dashed line: Michaelis-Menten function fit on data (R^2^ = 0.98). Each condition was tested in duplicate. (**D**) Slot-blotting of purified DNA. Left panel: UV-treated chromatin DNA (+UV) was strongly recognized by the DDB2 proteo-probe compared to untreated chromatin (no UV). Total DNA as a loading control was stained with methylene blue. Right panel: the DDB2 proteo-probe recognizes UV-irradiated plasmid DNA in a manner dependent on the amount of DNA. Hybridization of the DDB2 proteo-probe to the membrane was revealed by anti-FLAG immuno-blotting. (**E**) Flow cytometry analysis of untreated and UV-irradiated cells (30 J/m^2^ UV-C) using the DDB2 proteo-probe. a.f.u.: arbitrary fluorescence units. Dashed line: fluorescence threshold used to determine cells positively stained by the DDB2 proteo-probe: 7% and 55% of untreated and UV-irradiated cells, respectively (*P* = 5.77×10^−14^, two-sided Fisher's exact test).
(PDF)
