# Supplementary figures and images for: Monitoring Repair of UV-Induced 6-4-Photoproducts with a Purified DDB2 Protein Complex
Source: PLoS One. 2014 Jan 28;9(1):e85896. doi: 10.1371/journal.pone.0085896 (PMC3904869; doi:10.1371/journal.pone.0085896)

Figure S1

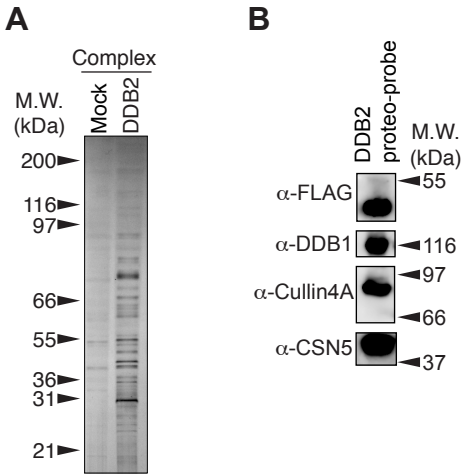

Supplement: Figure S1 — Analysis of the purified DDB2 protein complex components. (A) Visualization by silver staining of the DDB2 protein complex obtained by FLAG-affinity purification, and resolved by electrophoresis on a polyacrylamide gel. Purified DDB2 DNA damage recognition complex: “DDB2 proteo-probe”. M.W.: molecular weight; kDa: kiloDalton. (B) Western blotting analysis of key components of known DDB2 protein sub-complexes. DDB1 and Cullin4A of the ubiquitin ligase sub-complex as well as CSN5 of the COP9 signalosome sub-complex are detected along with FLAG-DDB2. (PDF) [file pone.0085896.s001.pdf]

Figure S2

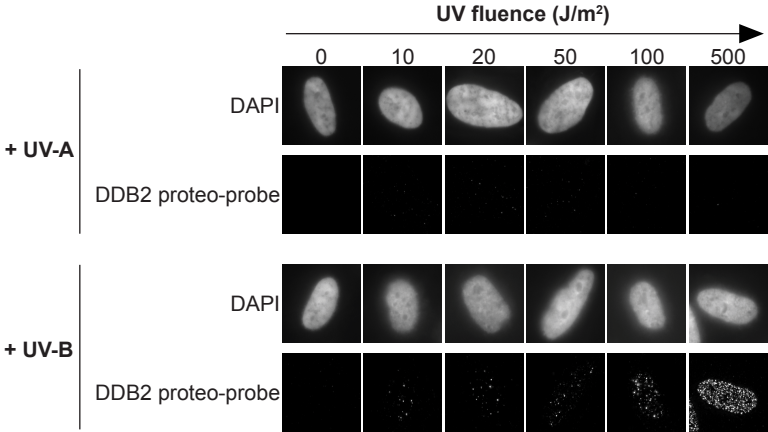

Supplement: Figure S2 — In situ detection of UV-A and UV-B DNA damage with the DDB2 proteo-probe. The DDB2 proteo-probe detects damage induced by UV-B but not UV-A. Fibroblasts were fixed prior to irradiation with different doses of UV-A or UV-B light. The DDB2 proteo-probe was added to fixed cells following irradiation. Hybridized DDB2 proteo-probe is revealed by anti-HA immunofluorescence. Nuclei are visualized by DAPI staining. One representative nucleus is shown for each experimental condition. (PDF) [file pone.0085896.s002.pdf]

**Figure S3**

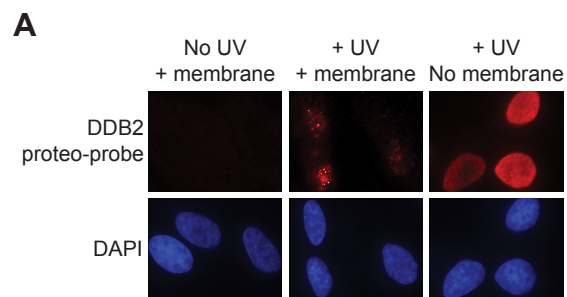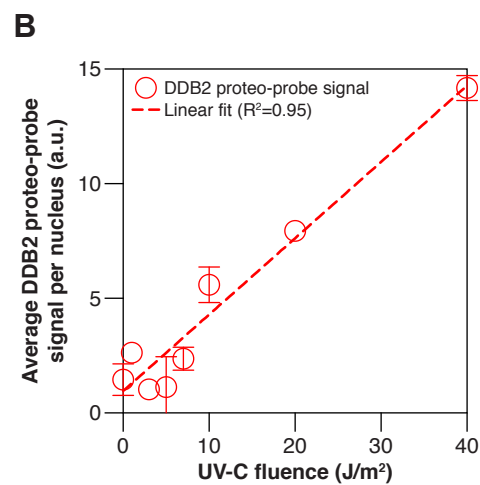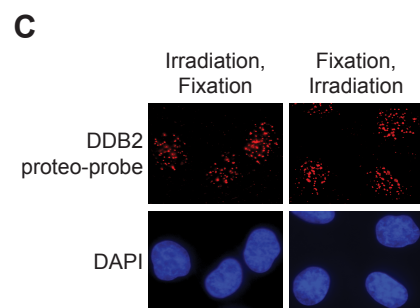

Supplement: Figure S3 — Characterization of the DDB2 proteo-probe hybridization properties. (A) The DDB2 proteo-probe signal is localized at sites of UV damage. Fibroblasts, uncovered or covered by a micro-porous membrane, were irradiated with UV-C (300 J/m2). (B) The DDB2 proteo-probe signal increases linearly with fluence. Fibroblasts were irradiated with different doses of UV-C. Each point is an average of three replicas. Each replica represents an average of at least 200 cells. Error bars: s.e.m. (C) The DDB2 proteo-probe signal is independent of endogenous proteins. Fibroblasts were irradiated with UV-C (10 J/m2), then fixed, or fixed then irradiated. The DDB2 proteo-probe was hybridized following fixation/irradiation. (PDF) [file pone.0085896.s003.pdf]

Figure S4

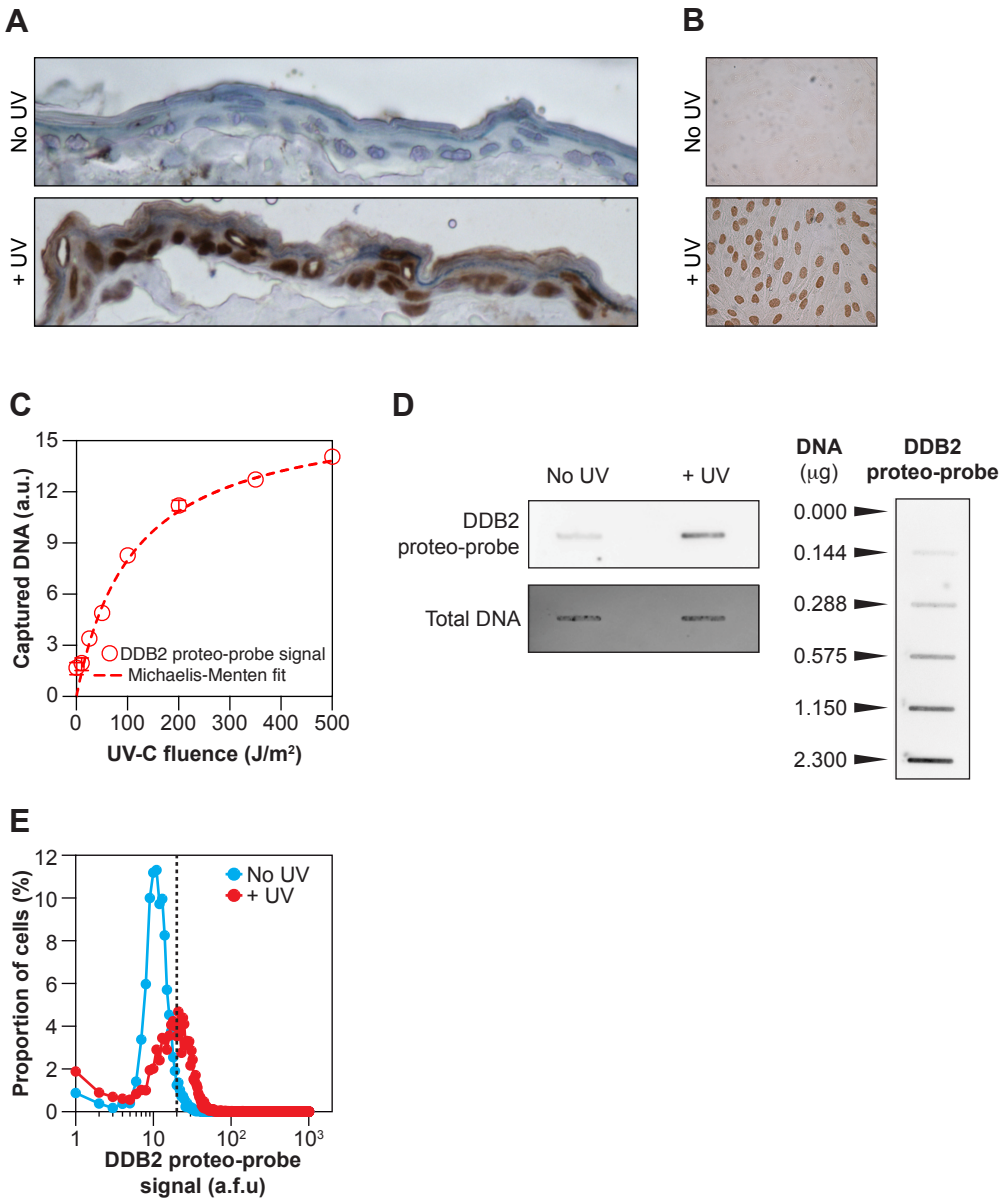

Supplement: Figure S4 — The DDB2 proteo-probe can be used in different assay formats. (A) Hybridization of the DDB2 proteo-probe onto frozen sections of irradiated mouse skin. The DDB2 proteo-probe bound to punch biopsies was revealed by HRP-conjugated anti-FLAG immunohistochemistry. (B) Irradiated fibroblasts (20 J/m2 UV-C) were fixed in methanol. Cytochemistry was done with the DDB2 proteo-probe in place of primary antibody. The hybridized proteo-probe was revealed by HRP-conjugated anti-FLAG. (C) The DDB2 proteo-probe retains irradiated plasmid DNA in a manner dependent on the amount of UV in an ELISA-like assay. Equal amount of the DDB2 proteo-probe was adsorbed onto wells of a 96-well microtiter plate. One hundred nanogram of UV-irradiated plasmid DNA was added in each well. Dashed line: Michaelis-Menten function fit on data (R2 = 0.98). Each condition was tested in duplicate. (D) Slot-blotting of purified DNA. Left panel: UV-treated chromatin DNA (+UV) was strongly recognized by the DDB2 proteo-probe compared to untreated chromatin (no UV). Total DNA as a loading control was stained with methylene blue. Right panel: the DDB2 proteo-probe recognizes UV-irradiated plasmid DNA in a manner dependent on the amount of DNA. Hybridization of the DDB2 proteo-probe to the membrane was revealed by anti-FLAG immuno-blotting. (E) Flow cytometry analysis of untreated and UV-irradiated cells (30 J/m2 UV-C) using the DDB2 proteo-probe. a.f.u.: arbitrary fluorescence units. Dashed line: fluorescence threshold used to determine cells positively stained by the DDB2 proteo-probe: 7% and 55% of untreated and UV-irradiated cells, respectively (P = 5.77×10^−14^, two-sided Fisher's exact test). (PDF) [file pone.0085896.s004.pdf]
